# How do people think about the implementation of speech and video recognition technology in emergency medical practice?

**DOI:** 10.1371/journal.pone.0275280

**Published:** 2022-09-23

**Authors:** Ki Hong Kim, Ki Jeong Hong, Sang Do Shin, Young Sun Ro, Kyoung Jun Song, Tae Han Kim, Jeong Ho Park, Joo Jeong

**Affiliations:** 1 Department of Emergency Medicine, Seoul National University Hospital, Seoul, Republic of Korea; 2 Department of Emergency Medicine, Seoul National University College of Medicine, Seoul, Republic of Korea; 3 Laboratory of Emergency Medical Services, Biomedical Research Institute, Seoul National University Hospital, Seoul, Republic of Korea; 4 Department of Emergency Medicine, Seoul National University Boramae Medical Center, Seoul, Republic of Korea; 5 Department of Emergency Medicine, Seoul National University Bundang Hospital, Seoul, Republic of Korea; Sri Eshwar College of Engineering, INDIA

## Abstract

**Background:**

Recently, speech and video information recognition technology (SVRT) has developed rapidly. Introducing SVRT into the emergency medical practice process may lead to improvements in health care. The purpose of this study was to evaluate the level of acceptance of SVRT among patients, caregivers and emergency medical staff.

**Methods:**

Structured questionnaires were developed for the patient or caregiver group and the emergency medical staff group. The survey was performed in one tertiary academic hospital emergency department. Questions were optimized for each specific group, and responses were provided mostly using Likert 5-scales. Additional multivariable logistic regression analyses for the whole cohort and subgroups were conducted to calculate odds ratios (OR) and confidence intervals (CI) to examine the association between individual characteristics and SVRT acceptance.

**Results:**

Of 264 participants, respondents demonstrated a positive attitude and acceptance toward SVRT and artificial intelligence (AI) in future; 179 (67.8%) for video recordings, and 190 (72.0%) for speech recordings. A multivariable logistic regression model revealed that several factors were associated with acceptance of SVRT in emergency medical practice: belief in health care improvement by signal analysis technology (OR, 95% CIs: 2.48 (1.15–5.42)) and AI (OR, 95% CIs: 1.70 (0.91–3.17)), reliability of AI application in emergency medicine (OR, 95% CIs: 2.36 (1.28–4.35)) and the security of personal information (OR, 95% CIs: 1.98 (1.10–3.63)).

**Conclusion:**

A high level of acceptance toward SVRT has been shown in patients or caregivers, and it also appears to be associated with positive attitudes toward new technology, AI and security of personal information.

## Introduction

In recent decades, speech and video information recognition technology (SVRT) using computerized data processing has developed rapidly [1, 2]. Machine learning and deep learning technology based on big-data make it possible to recognize speech and translate it into text information. SVRT can also be used to manage video figures, including human postures and skeletons [3, 4]. Several studies have found that current speech recognition technology has high levels of efficacy, accuracy and usability with respect to medical records [5, 6].

The implementation of SVRT has been shown to be useful in various aspects of medical practice [7–10]. In emergency medical practice, SVRT may also provide crucial benefits. First, medical records can be automatically generated without keyboard typing, thus decreasing the recording time by medical staff and increasing the accuracy level by eliminating typos [5, 11]. Medical records are important for organizing clinical information or communicating between medical providers, but the promptness and accuracy of manual typing by physicians have been questioned [12, 13]. Second, clinical data generated by SVRT could be used for the development of artificial intelligence (AI) -based clinical decision support. Innovative clinical tools can be developed based on large databases using speech and video information [10].

To develop and implement SVRT in routine emergency care, the acceptance of new technology by patients, caregivers and health care providers is essential. It is necessary to assess the level of acceptance by users and subjects prior to applying new technology in the clinical field [14]. For this purpose, several surveys have been conducted to examine the use of robots, telemedicine or general AI technology [15–17], but the acceptance of SVRT for emergency care, which is time sensitive and critical, has not yet been widely studied.

The purpose of this study was to evaluate the level of acceptance toward SVRT among patients or caregivers who visit the emergency department and emergency care providers. We hypothesized that the results would provide a basis for the successful implementation of new technology by identifying significant concerns and barriers.

## Methods

### Study design and setting

We designed a survey to study the acceptance of SVRT in emergency care among patients, caregivers and emergency care providers. This study was conducted in the emergency department (ED) of a tertiary academic hospital. This ED receives approximately 70,000 annual visits, and the department has approximately 10–12 attending physicians, 9–11 emergency medicine residents, 10 primary physicians, 50–60 emergency nursing staff and 15–17 emergency medical technicians.

### Participant selection and data collection

This survey used two types of standardized questionnaires optimized for patients/caregivers and emergency care providers. The survey was conducted between November 2018 and August 2019. The questionnaire was distributed to patients and caregivers who visited the ED for any cause by convenient sampling. This survey was conducted by face-to-face interviews using a paper questionnaire in an academic tertiary emergency department. Two emergency medical technicians who have experience in the emergency field were the research coordinators. They screened adult patients who were waiting for laboratory and radiographic test results as candidates with duty physicians. Recruitment was carried out while making sure to not interfere with the ED process, adversely affect the emotion of the patient and caregiver, or limit communication. Patients who were medically stabilized with sufficient cognitive function and caregivers of patients who were not in urgent status, were recruited to participate.

Concise introduction about SVRT was also given before the beginning of the survey. Coordinators waited nearby until they completed the questionnaire and answered any questions during the survey. They introduced the purpose of the survey very carefully and politely and immediately stopped if the participant refused to respond at any time. ED staff, including nurses, emergency medical technicians and physicians, were asked to participate in the survey after daywork by research coordinators. They voluntarily participated after explanations from researchers who had no hierarchical relationship in their duties.

### Questionnaire

The questionnaire consisted of 3 sections related to the acceptance of SVRT in emergency care: 1) prior knowledge and attitude, 2) acceptance toward automatic recording SVRT in emergency medical practice, and 3) demographic information of responders.

In the first section, we asked about prior knowledge and attitudes about SVRTs. Prior knowledge was evaluated differently between respondent groups. Patients and caregiver groups have more limited experience and knowledge about the medical field than emergency providers. To evaluate the attitude of respondents toward SVRTs, various questions were developed in the context of new technology, including AI in health care services and emergency medical fields. We asked about how they feel about recent trends of SVRT development. Five questions (3–5, 7–1, 8 in S1 and 4–6, 7–1, 8–1 in S2) were developed to evaluate perspectives about applicability and contribution. Since the attitude toward the competency of computer was considered a potent factor determining level of acceptance of participants, two questions were presented in a different way (7–2, 7–3 in S1 and 6 in S2). Additionally, questions measuring concern about personal information security were included (7–2, 7–3, 7–4 in S2).

In the second section, we evaluated acceptance toward automatic SVRT in emergency medical practice, assumed to be available in the near future. We introduced an a simulated SVRT system put in place for the purposes of this study in the study institution. In the triage process, speech and video information was collected by video cameras, speech recorders and data-saving computers based on the informed consent of patients and health care providers. Full interviews and physical examinations between emergency nurses and patients were recorded automatically. We asked about consent or rejection toward receiving medical practice with recording speech and video data. Further questions about detail concerns and discomfort were described as free text.

In the last section, we collected demographic information of respondents, including age, gender, education level defined as last diploma, affinity for computer technology, career in health care area, morbidity and frequency of ED visits. For the emergency medical staff version, questions were age, gender, affinity for computers, career in the medical field and profession they work in the ED.

Questionnaires were categorized into 2 versions according to the type of respondents–i.e., the emergency medical staff group and the patient and caregiver group. All details of both versions of the questionnaire are included in (S1 File) and (S2 File). Each version was optimized for the details of the specific subject group and was focused on the applicability of SVRT in real practice. Responses were mostly measured by a five-point Likert scale (adjusted for characteristics of each question) and multiple choice. For several open questions about concern, a text answer sheet was presented for description.

### Data analysis

Descriptive analyses of participant characteristics and answers were performed. Chi-square and Wilcoxon rank sum tests were used to compare the characteristics of the two groups. Respondents’ answers were measured by a 5-point Likert scale, and agreement regarding acceptance was transformed into binary variables for further analysis (strongly agree, agree as positive and neutral, disagree, strongly disagree as negative). A multivariable logistic regression model was developed to evaluate predictors of acceptance or refusal toward SVRT. Odds ratios (ORs) and confidence intervals (CIs) were calculated. To make a comprehensive model including both groups, variables are modified in the integrative method. We categorized most characteristics as binary variables: old age, defined as age 35 years and older, education above university graduate and experience of previous ED visit. Additionally, prior awareness was considered positive unless the participant denied and answered questions on a Likert-5 scale. In the scoring question about attitudes toward computer competency, we defined a score over 65 (two-thirds) as positive. Statistical analysis was conducted using R 3.5.2 with R Studio 1.1.463, and a p value <0.05 was considered statistically significant.

### Ethical approval

This study was approved by the institutional review board (IRB) of the study institution (IRB No. 1810-068-979). Documented informed consent was waived since the documenting questionnaire was considered to be in agreement.

## Results

### Characteristics of study population

Approximately 270 patients or caregivers and 50 ED staff were asked to complete a questionnaire, and approximately 50 patients or caregivers and 2 ED staff refused to respond. A total of 264 respondents were enrolled in the analysis (Patient or caregiver: 216, Emergency medical staff: 48). The emergency medical staff participants were more likely to be younger and had more education than the patients or caregivers. The proportion of subjects with computer affinity above fair was 66.2% in the patient or caregiver group compared to 73.0% in the emergency medical staff group (Table 1).

**Table 1:** Demographics and characteristics of study participants.

		Patient or caregiver	Emergency medical staff	
		N (%)	N (%)	p value
Total		216	48	
Female		130 (60.2)	25 (52.1)	0.39
Age, years				<0.01
	18–24	27 (12.5)	2 (4.2)-	
	25–34	81 (37.5)	33 (68.8)	
	35–44	63 (29.2)	13 (27.1)-	
	45–54	34 (15.7)	-	
	55–64	6 (2.8)	-	
	65-	5 (2.3)	-	
Education				<0.01
	Over University	136 (63.0)	48 (100.0)-	
Computer friendly				0.06
	Very poor	9 (4.2)	0 (0)	
	Poor	64 (29.6)	13 (27.1)	
	Fair	86 (39.8)	25 (52.1)	
	Good	57 (26.4)	9 (18.8)	
	Excellent	0 (0)	1 (2.1)	
Working experience in medical field		20 (9.3)	48 (100.0)	<0.01
Chronic disease		40 (18.5)	-	
Frequency of ER visit in last 6 month				
	0	159 (73.6)	-	
	1–3	52 (24.1)	-	
	4–6	5 (2.3)	-	
Career in medical field, years		-	5.1 (4.0)	
Profession in emergency department				
	EMT	-	11 (22.9)	
	Nurse	-	13 (27.1)	
	Doctor	-	9 (18.8)	
	Emergency physician	-	15 (31.2)	

SD, Standard deviation; ER, Emergency room; EMT, Emergency medical technician

### Prior knowledge and attitude about SVRT

Table 2 summarizes answers related to prior knowledge and attitudes about SVRTs, which was the first section of the questionnaire. Approximately half of the patient or caregiver group had heard about SVRT, and 29.2% of the emergency medical staff group had not heard about it. Respondents generally referred about speech recognition application in smartphone or human posture recognition games using Kinect. Both groups showed similar patterns of answers to questions about attitudes toward the rapid development of SVRT. In subjective free-text answers, 7 respondents described concerns about personal information issues. Above 70% of respondents agreed about the potential benefits of SVRT in health care services. The proportions were also similar in questions about physiologic signal analysis technology and AI applications in emergency medical practice.

**Table 2:** Prior knowledge and attitude toward speech and video recognition technology.

		Patient or caregiver	Emergency medical staff	
Variables		N (%)	N (%)	p value
	Total	216	48	
Prior awareness of SVRT				
	Yes	112 (51.9)	34 (70.8)	<0.01
Prior awareness of SVRT applied in medical field				
	Extreme aware	-	1 (2.1)	
	Very aware	-	5 (10.4)	
	Moderate aware	-	10 (20.8)	
	Slightly aware	-	20 (41.7)	
	Not at all aware	-	12 (25.0)	
Attitude toward rapid development of SVRT				0.53
	Completely satisfied	39 (18.1)	7 (14.6)	
	Very satisfied	87 (40.3)	18 (37.5)	
	Moderately satisfied	77 (35.6)	17 (35.4)	
	Slightly satisfied	12 (5.6)	5 (10.4)	
	Not at all satisfied	1 (0.5)	1 (2.1)	
SVRT can improve health care service level				0.84
	Strongly agree	50 (23.1)	14 (29.2)	
	Agree	111 (51.4)	22 (45.8)	
	Neither agree nor disagree	50 (23.1)	11 (22.9)	
	Disagree	5 (2.3)	1 (2.1)	
New technology analyzing physiologic signals can improve health care service level				0.42
	Strongly agree	64 (29.6)	20 (41.7)	
	Agree	115 (53.2)	20 (41.7)	
	Neither agree nor disagree	33 (15.3)	7 (14.6)	
	Disagree	4 (1.9)	1 (2.1)	
SVRT can improve human health and well-being				0.88
	Strongly agree	71 (32.9)	13 (27.1)	
	Agree	98 (45.4)	25 (52.1)	
	Neither agree nor disagree	39 (18.1)	9 (18.8)	
	Disagree	7 (3.2)	1 (2.1)	
	Strongly disagree	1 (0.5)	0 (0)	
AI can be applied in emergency medical field				0.35
	Strongly agree	53 (24.5)	11 (22.9)	
	Agree	96 (44.4)	24 (50.0)	
	Neither agree nor disagree	56 (25.9)	9 (18.8)	
	Disagree	7 (3.2)	4 (8.3)	
	Strongly disagree	4 (1.9)	0 (0)	
Human should have responsibility of decision with AI support				0.47
	Strongly agree	91 (42.1)	26 (54.2)	
	Agree	86 (39.8)	14 (29.2)	
	Neither agree nor disagree	30 (13.9)	6 (12.5)	
	Disagree	9 (4.2)	2 (4.2)	
Reliability level of decision by computer, Score (SD)		62.3 (18.6)	60.6 (16.7)	0.55
automatic medical record device improves health care service				0.12
	Strongly agree	43 (19.9)	4 (8.3)	
	Agree	103 (47.7)	21 (43.8)	
	Neither agree nor disagree	55 (25.5)	20 (41.7)	
	Disagree	12 (5.6)	3 (6.2)	
	Strongly disagree	3 (1.4)	0 (0)	
Hospital can prevent leakage of personal information				0.11
	Strongly agree	23 (10.6)	5 (10.4)	
	Agree	72 (33.3)	11 (22.9)	
	Neither agree nor disagree	94 (43.5)	21 (43.8)	
	Disagree	22 (10.2)	11 (22.9)	
	Strongly disagree	5 (2.3)	0 (0)	
Want to check speech and video data from medical practice				
	Strongly agree	79 (36.6)	-	
	Agree	81 (37.5)	-	
	Neither agree nor disagree	40 (18.5)	-	
	Disagree	14 (6.5)	-	
	Strongly disagree	2 (0.9)	-	
Want to possess speech and video data from medical practice				
	Strongly agree	59 (27.3)	-	
	Agree	68 (31.5)	-	
	Neither agree nor disagree	58 (26.9)	-	
	Disagree	23 (10.6)	-	
	Strongly disagree	8 (3.7)	-	

SVRT, Speech and video recognition technology; AI, Artificial intelligence; SD, Standard deviation

**Fig 1:**
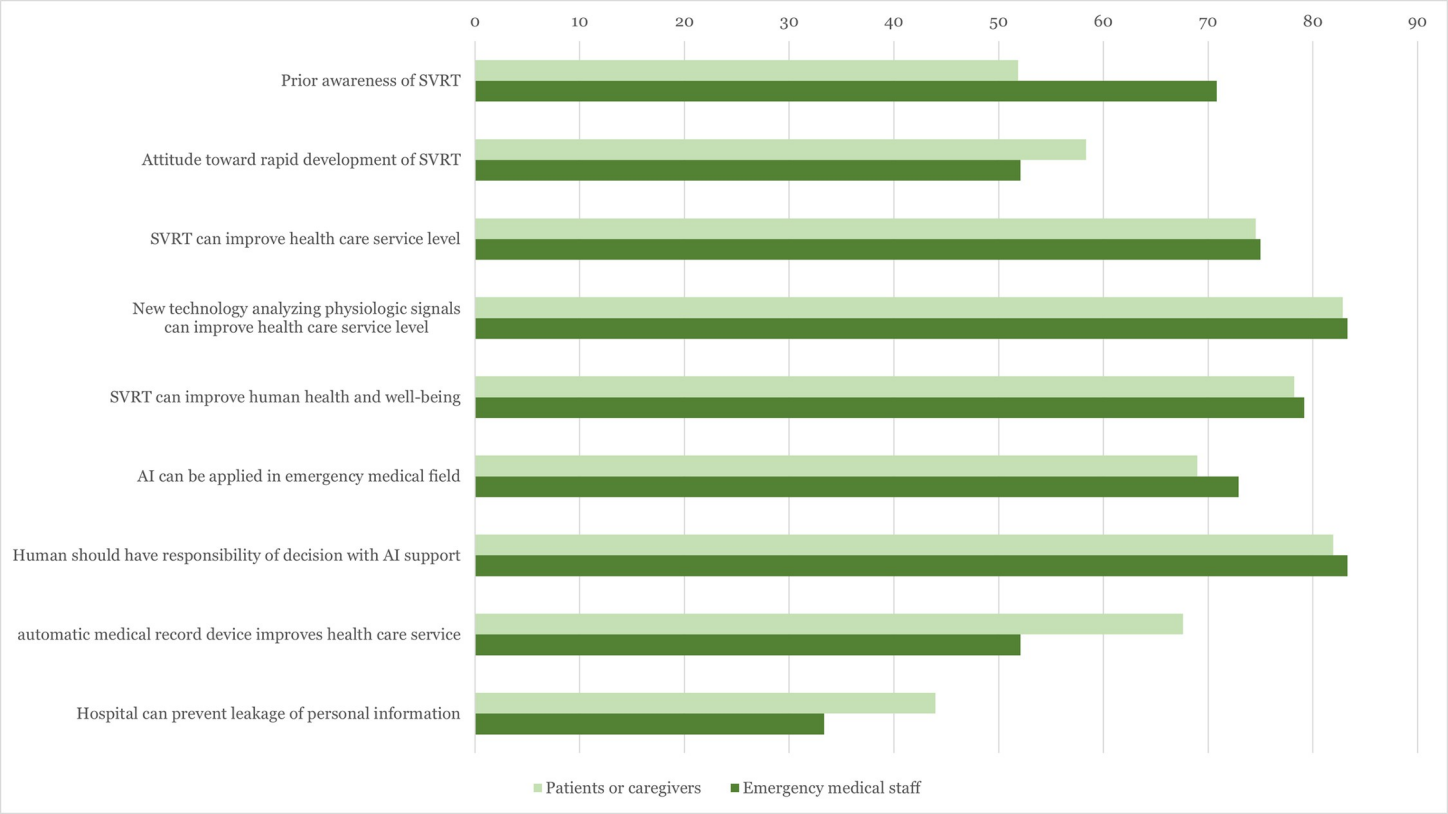
Proportion of positive response about prior knowledge and attitude toward SVRT according to study group. SVRT, speech and video recognition technology; AI, artificial intelligence.

Most respondents agreed that physicians are responsible for medical decisions, even though AI can support the physicians. The mean score for reliability level of medical decision by computer was 62.3 in the patient or caregiver group and 60.6 in the emergency medical staff group. Agreement toward the potential benefits of the automatic medical record system was relatively lower (67.6% in the patient or caregiver group, 52.1% in the emergency medical staff group). The proportion of respondents who believed the hospital could secure personal information was less than 50% in both groups (43.9% in the patient and caregiver group, 33.3% in the emergency medical staff group) (Fig 1).

### Acceptance toward SVRT

Regarding questions about speech and video recognition in emergency medical practice, 179 (67.8%) showed acceptance toward video recordings, and 190 (72.0%) toward speech recordings. The acceptance rate was higher in the patient or caregiver group than in the emergency medical staff group (71.8% vs. 50.0% in video recording, 76.9% vs. 50.0% in speech recording). Approximately half of the emergency medical staff group reported that they recommend the use of SVRT in the ED to relatives who require emergency care (Table 3). Various answers were described for subjective concerns about SVRT and application in emergency medical practice as below.

- Most people are not prepared to accept SVRT, and it cannot be used to improve the level of service
- Willing to welcome development in medical technology
- Wonder if there is any solution for the issue of personal information leaks
- AI is not thought to be an alternative for human medical providers
- Concern about insincerity in medical practice after refusing recording
- Concern about the abuse of personal data

**Table 3:** Acceptance toward video and speech recording in emergency medical practice.

		Patient or caregiver	Emergency medical staff
Variables		N (%)	N (%)
	Total	216	48
Acceptance toward video recognition in emergency medical practice			
	Yes	155 (71.8)	24 (50.0)
Acceptance toward speech recognition in emergency medical practice			
	Yes	166 (76.9)	24 (50.0)
Recommend SVRT in the ED to relative			
	Strongly agree	-	5 (10.4)
	Agree	-	19 (39.6)
	Neither agree nor disagree	-	11 (22.9)
	Disagree	-	9 (18.8)
	Strongly disagree	-	4 (8.3)
Feel okay about saving videos in ED		157 (72.7)	-
Feel okay about saving speech in ED		172 (79.6)	-

ED, Emergency department; SVRT, Speech and video recognition technology

### Multivariable logistic regression analysis

**Fig 2:**
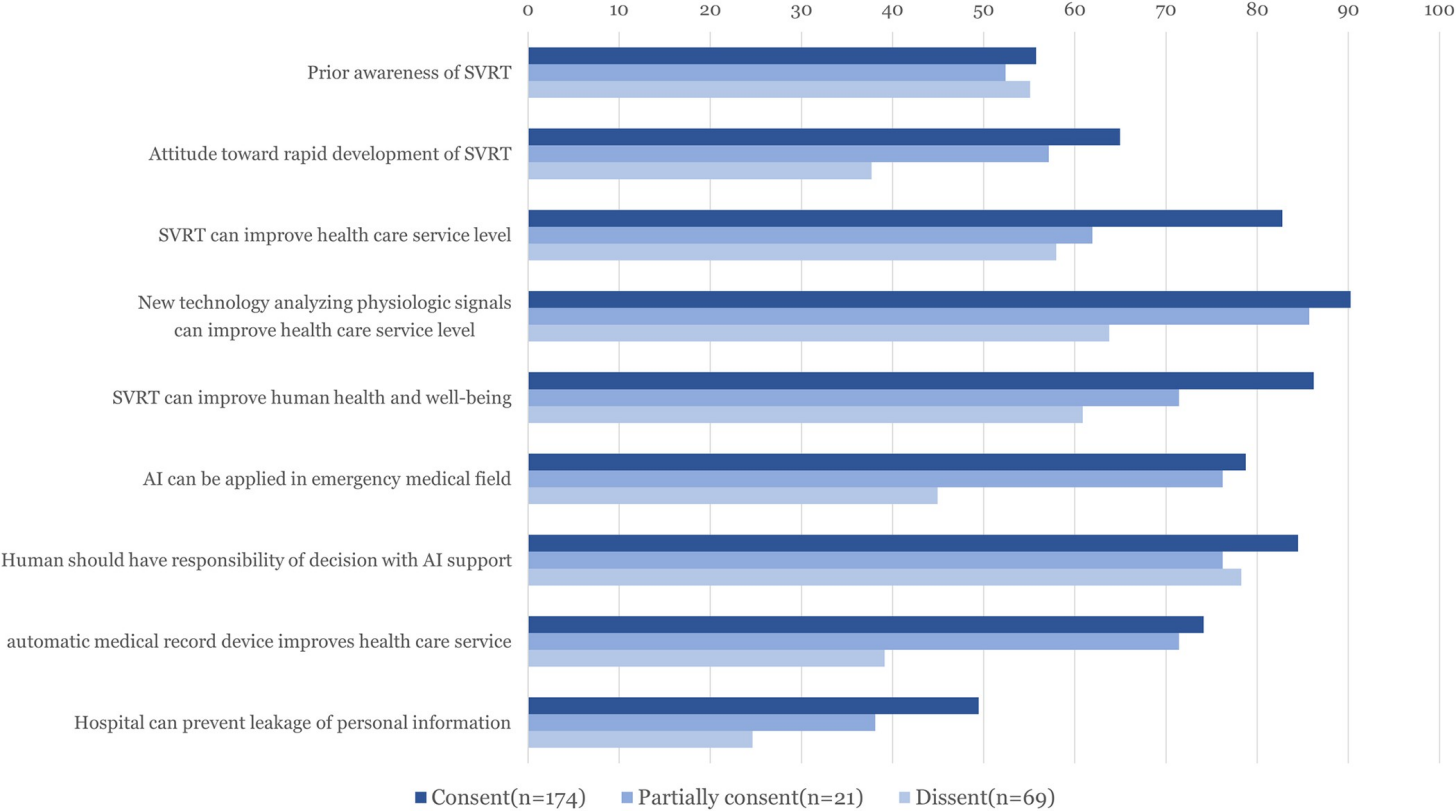
Proportion of positive response about prior knowledge and attitude toward SVRT according to acceptance. SVRT, speech and video recognition technology; AI, artificial intelligence.

We performed multivariate logistic regression to examine the acceptance of SVRT. In the stepwise model, five characteristics were selected: medical field experience (OR, 95% CI: 0.53 (0.29–0.99)), belief in health care service improvement by signal analysis technology (OR, 95% CI: 2.48 (1.15–5.42)) and AI (OR, 95% CI: 1.70 (0.91–3.17)), reliability of AI application in emergency medicine (OR, 95% CI: 2.36 (1.28–4.35)) and the security of personal information (OR, 95% CI: 1.98 (1.10–3.63)) (Table 4). Additionally, prior knowledge and attitude toward SVRT according to the acceptance for recording–consent for speech and video recording, consent either speech or video, or dissent—was compared (Fig 2).

**Table 4:** Multivariable logistic regression analysis for acceptance toward speech and video recording in emergency medical practice.

		Model	Stepwise model
Characteristics		Adjusted OR (95% CI)	Adjusted OR (95% CI)
Demographics			
	Age 35 years old and older	0.95 (0.53–1.71)	-
	Male gender	0.93 (0.52–1.67)	-
	Computer friendly	0.77 (0.40–1.46)	-
	University-level education	1.19 (0.59–2.39)	-
	Experienced in medical field	0.48 (0.24–0.95)	0.53 (0.29–0.99)
Prior knowledge and attitude			
	Prior knowledge of SVRT	1.18 (0.63–2.19)	-
	Positive attitude toward the rapid development of SVRT	1.36 (0.71–2.61)	-
Beliefs and thoughts			
	SVRT can enhance health care	1.09 (0.46–2.49)	-
	Signal analyzing technology can enhance health care	1.93 (0.72–5.18)	2.48 (1.15–5.42)
	SVRT can enhance human health	1.69 (0.72–3.95)	-
	AI can be applied in emergency medicine	2.06 (1.07–4.00)	2.36 (1.28–4.35)
	Humans should confirm medical decisions	0.92 (0.41–2.02)	-
	Reliability of decision by computer over 2/3	0.79 (0.41–1.49)	-
	AI can enhance health care	1.7 (0.88–3.27)	1.7 (0.91–3.17)
	Hospitals can prevent personal information leakage	1.79 (0.97–3.35)	1.98 (1.1–3.63)

OR, odds ratio; CI, confidence interval; SVRT, speech and video recognition technology; AI, artificial intelligence

Sensitivity analysis for patient or caregiver groups was conducted using the same method. Pervious ED visits (OR, 95% CI: 1.76 (0.84–3.90)), beliefs about health care service improvement by signal analysis technology (OR, 95% CI: 2.94 (1.32–6.64)), reliability of AI application in emergency medicine (OR, 95% CI: 2.87 (1.47–5.61)) and the security of personal information (OR, 95% CI: 2.03 (1.06–3.99)) were selected as predictors in the stepwise model (Table 5).

**Table 5:** Multivariable logistic regression analysis for acceptance toward speech and video recording in emergency medical practice in patients or caregivers.

		Model	Stepwise model
Characteristics		Adjusted OR (95% CI)	Adjusted OR (95% CI)
Demographics			
	Age 35 years old and older	1.11 (0.56–2.20)	-
	Male gender	1.21 (0.60–2.48)	-
	Computer friendly	0.95 (0.44–2.00)	-
	University-level education	1.25 (0.60–2.60)	-
	Experienced in medical field	1.63 (0.50–6.08)	-
	Chronic disease	0.95 (0.40–2.37)	-
	Recent ED visit	1.82 (0.81–4.33)	1.76 (0.84–3.90)
Prior knowledge and attitude			
	Prior knowledge of SVRT	1.11 (0.54–2.29)	-
	Positive attitudes toward the rapid development of SVRT	0.96 (0.43–2.07)	-
Beliefs and thoughts			
	SVRT can enhance health care	1.40 (0.52–3.70)	-
	Signal analyzing technology can enhance health care	1.76 (0.57–5.41)	2.94 (1.32–6.64)
	SVRT can enhance human health	1.71 (0.63–4.60)	-
	AI can be applied in emergency medicine	2.63 (1.22–5.73)	2.87 (1.47–5.61)
	Humans should confirm medical decisions	0.83 (0.33–2.02)	-
	Reliability of decision by computer over 2/3	0.69 (0.32–1.46)	-
	AI can enhance health care	1.46 (0.63–3.31)	-
	Hospitals can prevent personal information leakage	1.77 (0.86–3.73)	2.03 (1.06–3.99)
Request about data			
	Check after recording	0.78 (0.29–2.01)	-
	Keep after recording	1.33 (0.56–3.08)	-

OR, odds ratio; CI, confidence interval; ED, emergency department; SVRT, speech and video recognition technology; AI, artificial intelligence

## Discussion

We tried to determine the acceptance of SVRTs by laypersons who visited EDs and health care providers working at EDs through a survey with a structured questionnaire. The majority of respondents showed a positive attitude toward SVRT application in emergency medical practice. There was a higher acceptance rate in the patient or caregiver group than in the emergency medical staff group. The questionnaire revealed that several factors were associated with acceptance of SVRT, including experience with the medical field, belief in health care service improvement by signal analysis technology and AI, reliability of AI application in emergency medicine, and the security of personal information.

This study is the first survey aimed at evaluating level of acceptance and attitude toward SVRT in the emergency medical field. We categorized the main dimensions as prior knowledge, attitude toward SVRT and acceptance toward implementing SVRT in emergency care. Unlike previous studies on patient satisfaction or experience [18, 19], this survey was focused on personal beliefs and compliance with new technology using speech and video, which is not currently used in practice.

In our results, trust in new technologies and guarantee of security for personal information seem to be crucial in the level of acceptance of SVRTs in emergency practice. Contrary to our concerns, some studies have shown that people are less concerned about personal information leakage when communicating with AI [20, 21]. It can be estimated that the level of trust toward new technology has an inverse relationship with anxiety about personal information security. Thus, trust can be fostered by establishing a higher level of privacy when new technologies are applied. Interestingly, this trust level was not seemed to be associated with younger age or computer affinity according to the regression model in the study. It may be just characteristics of the emergency medical staff group, but it should be considered that the regional demographics and social characteristics may affect the consent rate of new technology implementation.

We found that emergency medical staff reported more negative attitudes overall than patients and caregivers. This is a notable finding, as a clinical trial-related survey study showed the opposite result [22]. It is assumed that automated recording from medical practice does not always seem beneficial for medical staff. Magowan et al. demonstrated that many surgeons felt uncomfortable when they were asked to be recorded during surgery [23]. Extracting detail information and assisting to facilitate the medical practice process are separate issues. Many doctors may feel most of information from automated recording as false alarm from monitor and would not change patient care [24]. We can also infer that medical staff are quite reluctant to expose information related to their role performance, even more than the patients exposing themselves. For these reasons, it is necessary to consider the risk of the medical staff’s treatment behavior becoming passive with the SVRT system. This is a recent growing issue in the area of patient recording clinic visits [25].

Implementation of speech and video recording in emergency medical practice by new technology development is acceptable in patients or caregivers. Researchers who tried to develop technology recognizing comprehensive information in the medical field may consider what respondents feared and were concerned about. It is necessary to discuss various fears and build positive attitudes when new technology is introduced [26]. Most of the free-text answers in questionnaires are about distrust about the performance of AI itself rather than speech and video recognition. Although considerable research has been conducted using artificial technology in medical practice, practical benefits are not clear [27]. Further efforts to clarify the superiority and beneficial effects of AI are crucial. There was also concern about the association between technology improvement and practical benefits that patients can obtain.

Fear about personal information leaks has also been shown to be an important factor for acceptance. Recently, access and disclosure of personal health information have been considered important dimensions in medical practice [28]. To assure respondents about this issue, robust and accredited protocols and methods should be prepared. Technical methods to prevent privacy issues are crucial, such as blurring faces or using polygon meshes in video recognition [29].

There are several limitations in the study. First, several questions had different answer formats across the groups. We assumed a fundamental difference in the level of background knowledge between the general population and medical staff about new technologies in the medical field. Question asking whether want to check or possess the data were not included in the questionnaire for medical staff, since they didn’t seem to be interested. Second, we categorized patients and caregiver as the same group, but there could be different characteristics and attitude, according to the discomfort or pain. Although we could request the survey only for stable patients, this is also a significant limitation. Third, this study was conducted in one tertiary academic hospital emergency department and was a convenience sample. Also, we necessarily excluded patients who were unstable, or caregivers of unstable patients in the acute care setting, which may have biased our results. To generalize the results, further multi-center studies are needed. Next, our work is a first attempt to evaluate attitudes and acceptance toward SVRT, but a robust qualitative assessment should be conducted among populations with specific diseases. For example, time-sensitive emergency diseases could benefit from the use of SVRT in the ED by shortening the medical record typing time, but herein disease entity and severity were not considered in candidate selection for questionnaires. Finally, the questionnaire in this study was composed of mostly of positive questions, which may have influenced the responses. We are designing future surveys that will include both positive and negative questions.

## Conclusion

This study showed that the patient or caregiver group and emergency medical staff group were acceptable for implementing SVRT in emergency care. Positive acceptance of SVRT in emergency care was associated with positive attitudes toward belief in health care service improvement by signal analysis technology and AI, reliability of AI application in emergency medicine and the security of personal information. To implement new technology in the emergency practice, demonstrating strict regulation and protocol for personal information security should take precedence.

## Supporting information

S1 File The questionnaire for emergency medical staff. The questionnaire was administered to emergency medical staff who finished the duty to study their knowledge, attitudes and acceptance of speech video recognition technology in emergency medical practice.
(PDF)

S2 File The questionnaire for patient or caregiver. The questionnaire was administered to the patients or caregivers waiting for laboratory and radiographic tests in emergency departments to study their knowledge, attitude and acceptance of speech-video recognition technology in emergency medical practice.
(PDF)
