# Supplementary material for: How do people think about the implementation of speech and video recognition technology in emergency medical practice?
Source: PLoS One. 2022 Sep 23;17(9):e0275280. doi: 10.1371/journal.pone.0275280 (PMC9506645; doi:10.1371/journal.pone.0275280)
Supplement: S2 File — The questionnaire was administered to the patients or caregivers waiting for laboratory and radiographic tests in emergency departments to study their knowledge, attitude and acceptance of speech-video recognition technology in emergency medical practice. (PDF) [file pone.0275280.s002.pdf]

S2. The questionnaire for patient or caregiver

Our laboratory operates a designed studio for extracting speech and video data from medical history taking and physical examination between emergency physicians and patients. This studio does not interrupt the routine practice course of the emergency department, and there is no possibility of harm in medical process. It only records the first interview with the doctors, and identifiable personal information such as faces or names will be deleted or blurred.

The purpose of this studio is to construct a digital database of speech and video information from real medical practice in the emergency department. Based on the database, artificial intelligence technology such as machine learning is expected to support doctors' decision to diagnose urgent disease and start prompt treatment. The development of new medical database systems and decision support tools by artificial intelligence technology would ultimately improve the promptness and accuracy of diagnosis and treatment, which is expected to improve health care services for humans.

For few minutes, we would like you to do us a favor. This questionnaire was used to evaluate prior knowledge and acceptance toward implementing "speech and video recognition technology" in the medical practice of emergency departments. If there is any discomfort or interruption in your visit, please inform the research coordinator who requested this survey, and it will be discontinued immediately.

A. Prior knowledge and acceptance toward speech and video recognition technology (SVRT)

1. Were you aware of SVRT before this survey? (different)

   Yes □ No □

2. How do you feel about the recent rapid development of SVRT?

   ① Completely satisfied ② Very satisfied ③ Moderately satisfied ④ Slightly satisfied ⑤ Not at all satisfied

   2-1 Would you write down your opinion if you answered as not so satisfied at the above question.

----

3. Do you think the development of SVRT can improve the health care service level?

   ① Strongly agree ② Agree ③ Neither agree nor disagree ④ Disagree ⑤ Strongly disagree

4. Recent studies on new technology are evolving and enable the evaluation of patient status and prediction of danger by analyzing various physiological signals, such as electrocardiograms, electroencephalography, and blood pressure. Do you think that the development of this type of technology can improve health care service levels?

   ① Strongly agree ② Agree ③ Neither agree nor disagree ④ Disagree ⑤ Strongly disagree

5. Do you think applying new technology such as SVRT can be helpful in improving human health and well-being?

   ① Strongly agree ② Agree ③ Neither agree nor disagree ④ Disagree ⑤ Strongly disagree

6. In the medical field, artificial intelligence computer recognition technology is beginning to support various works, such as interpreting radiologic examinations or pathology slides, which have been dependent on medical providers.

   6-1. Do you think that human medical providers should have responsibility for final decisions regarding diagnosis and treatment events if artificial intelligence recognition computer technology is applied?

   ① Strongly agree ② Agree ③ Neither agree nor disagree ④ Disagree ⑤ Strongly disagree

6-2. How much do you trust about computers' decisions about diagnosis and treatment in human patients? Please fill in the box below

0% 10% 20% 30% 40% 50% 60% 70% 80% 90% 100%

7. Recently, in the clinic, ward or operating room, speech and video recordings have been performed in the earnest for rapid processing and secure medical records.

7-1. Do you think this kind of environmental change is associated with improvements in health care service levels?

① Strongly agree ② Agree ③ Neither agree nor disagree ④ Disagree ⑤ Strongly disagree

7-2. Do you think hospitals can prevent leakage of personal information by personal information protection protocols?

① Strongly agree ② Agree ③ Neither agree nor disagree ④ Disagree ⑤ Strongly disagree

7-3 Do you want to check your speech and video data from medical practice later?

① Strongly agree ② Agree ③ Neither agree nor disagree ④ Disagree ⑤ Strongly disagree

7-4 Do you want to possess your speech and video data from medical practice?

① Strongly agree ② Agree ③ Neither agree nor disagree ④ Disagree ⑤ Strongly disagree

8. Do you think that artificial intelligence recognition computer technology can be applied in emergency medical practice?

① Strongly agree ② Agree ③ Neither agree nor disagree ④ Disagree ⑤ Strongly disagree

B. Acceptance toward speech and video recording of medical practice in the emergency department

9. Will you accept video recording your medical practice when you are assigned to the SVRT applied space of emergency department?

Yes □ No □

10. Will you accept speech recording your medical practice when you are assigned to the SVRT applied space of emergency department?

Yes □ No □

11. Do you feel it’s okay to save your video?

Yes □ No □

12. Do you feel it’s okay to save your speech?

Yes □ No □

13. From your perspective, do you have any discomfort or concern about applying SVRT in emergency medical practice? Please write down in comfort.

C. Demographic findings

We would like to check basic information about your background. This information will not leak or be used for reasons other than research.

14. What is your gender?

Male □ Female □

15. How old are you? (different)

18-24 □ 25-34 □ 35-44 □ 45-54 □ 55-64 □ 65- □

16. What is your final education? (different)

Elementary school □ Middle school □ High school □ University □ Postgraduate □, etc. □

17. How well do you use computer?

Very poor □ poor □ Fair □ Good □ Excellent □

18. Are you working in medical field?

Yes □ (What is your job? _________) No □

19. Do you have chronic disease?

Yes □ No □

20. How many have you visited the emergency department in the last 6 months?

Today is the first day □ 1-3 □ 4-6 □ 7-9 □ More than 9 □
